# Knowledge and practice of exclusive breastfeeding among mothers in the tamale metropolis of Ghana

**DOI:** 10.1186/s12978-018-0579-3

**Published:** 2018-08-22

**Authors:** Ruth Nimota Nukpezah, Samuel Victor Nuvor, Jerry Ninnoni

**Affiliations:** 1 Department of Nursing, University for development studies, Tamale, Ghana; 2 School of Medical Sciences, University of Cape Coast, Cape Coast, Ghana; 3 School of Nursing and Midwifery, University of Cape Coast, Cape Coast, Ghana

**Keywords:** Exclusive breastfeeding, Knowledge, Practice, Tamale, Ghana

## Abstract

**Background:**

The prevalence of exclusive breastfeeding (EBF) for the first six months of life has remained low worldwide and in Ghana, despite strong evidence in support of its practice. This study was aimed at assessing the knowledge and practice of exclusive breastfeeding among mothers in the Tamale metropolis of Ghana.

**Methods:**

In a descriptive cross-sectional study, 393 mother-infant pairs attending child welfare clinics from three health facilities in the Tamale Metropolis were surveyed. A structured item questionnaire was used to collect data on the socio-demographic Characteristics of the participants, their knowledge regarding breastfeeding and level of practice of exclusive breastfeeding (EBF). The results were presented in frequency counts, percentages and inferences were made using a contingency table and chi-square values were computed to check for the relationship between participants demographic characteristics, the Knowledge and the practice of EBF and *P* value was set at 0.05.

**Results:**

The Analysis of the data was done with SPSS version 20. The study surveyed a total of 393 mothers from Tamale metropolis, of whom 27.7% reported having exclusively breastfed their infant for the first 6 months of life. The socio-demographic of the participants showed that they all had some level of education. The study revealed that 39.4% initiated breastfeeding within one hour after birth. Majority of participants had heard of EBF 277 (70.5%), about 344 (87.5%) of participants believed that EBF should be practised for 5 months in their locality. Pearson Chi-square test of the association between sociodemographic characteristics associated and EBF showed a significant association between EBF and the sex of the child, X^2^ = 4.177, *P* = .041. Whiles, EBF and the Knowledge on child spacing was X^2^ = 17.769, *P* < .001 and EBF and knowledge on Breast cancer reduction was also significant X^2^ = 4.384, *P* = .036.

**Conclusions:**

Although all the participants had some level of education background, a majority did not have adequate knowledge on EBF and EBF practice was low in the study community. Thus, we suggest improved education at the child welfare clinics and the media should be used as a platform to educate women adequately about importance of EBF.

## Plain English

The factors contributing to the reduced rate of EBF practice in developing countries such as those in Africa has been shown to include: lack of knowledge about benefits of breastfeeding, lack of maternal decision-making power, work schedules, recommencing work after maternity leave, lack of support, traditional beliefs and practices. To address the issue, this study explored the knowledge and practice of Exclusive breastfeeding among mothers attending post-natal clinics in the Tamale metropolis. In a descriptive cross-sectional study, 393 mother-infant pairs attending child welfare clinics from three health facilities in the Tamale Metropolis were surveyed.

A structured item questionnaire was used to collect data on the socio-demographic characteristics of the participants, knowledge of mother regarding breastfeeding and level of practice of exclusive breastfeeding. Data analysis was carried out with the aid of IBM Statistical Package for the Social Sciences (SPSS) version 20 for Windows and reported using, tables and prose. The study revealed that though the participants all had some level of education, a majority believed that EBF for a child should be less than 6 months after birth, hence, majority of the participants had inadequate knowledge on EBF. To add to this, the rate of EBF practice was found to be low in the study area.

## Background

Breast milk has the essential nutrients that a newborn need to grow healthy and strong. Infants who are exclusively breastfed develop fewer infections; have less severe illnesses and mothers who practice EBF enjoy the benefit of extended lactation amenorrhea [1]. Adequate knowledge about exclusive breastfeeding is said to be the fundamental tool that can direct the course of EBF practice among mothers [2, 3]. Yet [4], indicated that most mothers have knowledge of EBF 97.6%, but only 64.6% have adequate knowledge and mother’s higher knowledge about breastfeeding correlated with longer duration of practice. Meanwhile [5], in a study on knowledge of the importance of EBF for the first 6 months, also showed that the knowledge level of participants was (35.7%), this figure was seen to be relatively high. Yet, only about half of the participants (17.9%) practised EBF.

The risk of conditions such as, breast and ovarian cancer is lessened among mothers who adequately practice EBF [1]. World health organization indicated that EBF protective effect is not limited to the lactation period; it persists for years even after termination of breastfeeding. The benefits of EBF are bigger in settings of poverty, poor nutrition and poor hygiene, where baseline disease rates are higher. This is because giving babies other feds before six months is often associated with errors of contamination during the preparation and administration process. These errors can further lead to diarrheal diseases like cholera and dysentery which can culminate in childhood mortality [1, 5].

According to [6], with EBF coverage of 90%, about 13% of deaths of children less than 5 years could be averted in low and middle-income countries. This assertion is in line with other researchers who opined that initiation of breastfeeding within the first hour of birth may lead to the prevention of about 20% of neonatal deaths [7, 8] In low-income/middle-income countries. And optimal breastfeeding has the potential of preventing 12% of all under-5 deaths [9]. Children who are exclusively breast fed have been shown to be less susceptible to childhood diseases and are 14 times more likely to endure ill-health compared with those who are not breastfed [10].

Childhood mortality is high in low and middle-income countries where EBF prevalence is low. In Ghana for instance, the recorded rate of infant mortality is 53 per 1, 000 live births while mortality rate of children younger than 5 years is 31 per 1000 live births and these death ratios is partly due to inadequate EBF practice of mothers [11].

Other studies undertaken in Ghana also concluded that neonatal deaths could be prevented if all infants start breastfeeding within the first hour of birth [12].

Some of the interventions and policies introduced in Ghana to help encourage EBF practice are infant and young child feeding (IYCF) program, convention on the rights of the child and baby friendly Hospital initiative [6]. Despite the enactment of these policies, the rate of EBF in Ghana is still lower than the WHO's set goal of 90% for EBF. A report from Ghana multiple indicator cluster surveys showed that EBF in Ghana dropped drastically from 63.7% in 2008 to 46% in 2011 [12]. More specifically, the rate of EBF in Tamale was 63.3%. In effect, the rate of EBF is low globally (39%), with 36% occurring in low-income countries [1]. Several factors have been identified as impediments to proper nutrition and infant feeding habits. Substantial among them are, the perceptions surrounding infants feeding practices and inadequate information and support on good feeding practises, especially EBF for the first six months of life [13]. However, from the literature reviewed at the start of this study, there was no documented literature on the related factors associated with the knowledge and practice of EBF in the Tamale metropolis and no study had coved a vast majority of EBF among lactating mothers in the major Hospitals of the Region. This paper discusses the knowledge and practice of EBF among mothers visiting the child welfare clinics at Tamale West Hospital, Tamale Central Hospital and the Tamale Teaching Hospital in Ghana.

### Research methods

#### Study setting

Tamale is the capital of Northern Region of Ghana and the metropolitan assembly is one of the 20 administrative districts in the Region. The Metropolitan is divided into three Sub-Metros; Tamale North Sub-Metropolitan, Tamale Central Sub-Metropolitan and Tamale South Sub-Metropolitan. Tamale Teaching Hospital (TTH), Tamale West Hospital (TWH) and Tamale Central Hospital (TCH) are the three main public facilities in the Region. Tamale Teaching Hospital is a tertiary referral Centre for the three northern regions. Tamale West Hospital and TCH are secondary level service point. These health facilities provide maternal and neonatal Care services that enriched the study objectives.

### Population

The study populations were resident, lactating mothers of the Tamale Metropolitan area with a child of at least six months and at most two years at the time of the research. The participants attended child welfare clinic (CWC) in Tamale Teaching Hospital, Tamale West Hospital or Tamale Central Hospital. Using mothers who attend CWC at these facilities was useful to the study because these facilities are the main public facility in the region and has lots of client’s patronage at the CWC. The annual estimate of the population of women who attends CWC at the selected Hospitals in 2015 was: TTH = 5986, TWH = 2999 and TCH = 2995. Total = 17,960.

### Inclusion criteria

Lactating mothers who had children from ages 6 months - 2 years attending the Child Welfare Clinic (CWC) at Tamale Teaching Hospital, Tamale West Hospital and Tamale Central Hospital.

### Exclusion criteria

The study excluded lactating mothers having children with Medical Conditions that prevented the practice of EBF such galactosemia and mothers who were unwilling and mothers with children less than 6 months.

### Sampling procedure

#### Sample size determination

The desired sample was obtained using the Cochran’s formula for calculating sample size *n* = *z*^2^*pq*/*d*^2^ [14].

Where: n = desired minimal sample population.

z = standard normal deviate which is 1.96 at 95% confidence level.

*p* = 63.3% (2011 estimated figure of exclusive breastfeeding rate in Tamale [12]).

d = degree of accuracy 5%.

q = 1-p.

Hence substituting the value into the formula: *n* = *z*^2^*pq*/*d*^2^.

$$ n=\frac{(1.96)^2\left(0.633\times 0.367\right)}{(0.05)^2} $$

$$ n=356.97\approx 357 $$

To address the possibility of non-response/incomplete questionnaires and attrition, 20% was added to the calculated sample size.

Hence, a total sample size of 428 working mothers with infant pairs was estimated for the study.

### Sampling methods

Three types of sampling methods were used at different stages of the study. First and foremost, three Hospitals in Tamale Metropolis were purposively sampled. The Hospitals were: Tamale Teaching Hospital (TTH), Tamale West Hospital (TWH) and Tamale Central Hospital (TCH). These Hospitals were selected as these facilities are the major Hospital in the metropolis, the Hospitals serves as referral centers for clients from the surrounding district and the Hospitals had a child welfare clinic. Secondly, a quota sampling was used to calculate the sample size for each Facility [14]. Described quota sampling as a type of non-probability sampling where participants were selected non-randomly, based on their known proportion to the population.

The sample size for each facility was estimated proportional to their population size. We recruited an equal proportion of working mothers with infant’s pair from Tamale West Hospital and Tamale Central Hospital, but the proportion was doubled in Tamale Teaching Hospital because of the population size was twice of the Tamale West Hospital and Tamale Central Hospital respectively. Thus, TTH = 5986, TWH = 2999, TCH = 2995, summing up to 17,960 population size. Using, $Sample\kern0.5em fraction\kern0.5em (f)\kern0.5em =\kern0.5em \frac{sample\kern0.5em size\kern0.5em (n)}{targeted\kern0.5em population\kern0.5em (N)}$ [14].

This extrapolates to sample size of Tamale Teaching Hospital 214, the sample size of Tamale West Hospital 107, and 107 for Tamale Central Hospital. This gives a total of 428 sample size.

Thirdly, at the facility level, simple random Probability sampling method was used to select the participants. [14] indicated that probability sampling allowed researchers to make a generalization to the population defined by the sampling frame, whereas nonprobability sampling does not make room for generalization to be made beyond the sample. This assertion informed the researcher’s decision to use a simple random sampling technique to enlist the participants.

The participants were recruited by the researcher within eight (8) consecutive weeks in each of the health facilities. Simple random sampling (balloting) technique was utilized. On each clinic day, a list of mothers- infants pair who were registered for weighting and immunization was used as a sampling frame, they were assigned identification (ID) numbers. The numbers were listed in separate pieces of papers and placed into a bag. The papers were shuffled after which ballots were drawn from the bag without replacement. This exercise was repeated on each clinic day until the required sample of 428 (100%) participants was attained.

### Pretesting

The pilot study was conducted among 10 mother-infants pair of similar age (6 to 24 months) in Savelugu Hospital which is a neighbouring community. Based on the responses that were received, appropriate modifications were made to the questionnaires to ensure accuracy, clarity and reproducibility. In addition, Correlation coefficient to check for the instrument’s internal consistencies was also determined using SPSS Cronbach correlation formula^.^ The tools were accepted with the coefficient of 0.877.

### Data collection procedure

The research instrument used was a self-administered questionnaire. According to [14], questionnaire enhances greater privacy and reduces the incidence of bias responses which is often associated with other data of collection instrument such as interviews. The questionnaire consisted of two sections derived from the aim of the study: Knowledge and the practice of Exclusive breastfeeding.

Data were collected at the child welfare clinic of the selected hospitals and the mothers were given an average time of 15-min each to answer the questionnaires. About 428 questionnaires were given out. Only the 393 questionnaires which were completely answered were used for the final analysis. The data collection instrument (questionnaire) was in the English language. For participants who could not read well/understand some of the questions, the question items were read to them and appropriate options chosen by the clients was marked. The questionnaire comprised Yes/No questions on the level of practice of EBF and some categorical itemized questions about the participant's knowledge of EBF. The total number of questions were 25.

### Data processing and analysis

Data analysis was carried out in June with the aid of IBM Statistical Package for the Social Sciences (SPSS) version 20 for Windows and reported using, tables and prose. The process for the data analysis included; data coding, sorting, cleaning, editing and checking for errors and biases by doing a thorough counting of the study question items and frequency of occurrences, the grouping of collected data, checking for minimum and maximum counts and analysing and discussing of data. The questionnaire was given to my supervisors to review and the analysis that was done after the study was also given to them to cross-check for approval. A descriptive table was drawn to show the socio-demographic, practice of EBF and Knowledge of participants about EBF. Further inferences were made using a contingency table and chi-square values was computed to check for the relationship between participants demographic characteristics, the Knowledge and the practice of EBF and *P* value was set at 0.05.

## Results and discussion

The data were analysed using frequencies, percentages and prose. The analysis also included cross-tabulation and Pearson chi square test. Some responses were scored dichotomously as Yes = 1 and No = 0 and some questions were multi choice responses. Eight (8) knowledge questions and 8 demographic characteristics questions were presented respectively, whiles the practice Item questions were Nine (9). In carrying out this study, the researcher used a sample of 428 respondents and a total of 428 questionnaires was distributed to the lactating mothers. However, 393 questionnaires were returned fully answered with other questionnaires having 35 missing data. Therefore, the sample size for answering the research questions was 393 indicating 91.8% of total response rate.

### Results

#### Results on socio-demographic characteristics of participants

The distribution of demographic data of respondents involved in the study. From Table 1, the majority, 204 (51.9%) of the respondents were JHS level and lower, SHS were 100 (25.4%), and lastly, those who had attained tertiary levels were 89 (22.6%). The study, therefore, revealed that majority of the respondents involved in the study were having some form of education. Other sociodemographic characteristics of respondents are summarized in Table 1.

**Table 1:** Socio-demographic data of the respondents

Variables	Frequency	Percent (%)
Education of mother		
JHS and lower	204	51.9
SSS	100	25.4
Tertiary	89	22.6
Total	393	100.0
Form of employment		
Unemployed	137	34.9
Worker	256	65.1
Total	393	100.0
Monthly Income (Ghana cedi)		
Low: ≤ 1000	135	34.4
Middle: 1000–2000	209	53.1
High: ≥ 2000	49	12.5
Total	393	100.0
Age of mother		
≤ 25 years	239	60.8
≥ 25 years	154	39.2
Total	393	100.0
Total number of children		
1	142	36.1
≥ 1	251	63.9
Total	393	100.0
Place of birth		
Non-Health Facility	143	36.4
Health facility	250	63.6
Total	393	100.0
Sex of child		
Female	277	57.8
Male	166	42.2
Total	393	100.0
Age of child		
6–24 Months	209	53.2
6 months	184	46.8
Total	393	100.0

Source: Field data, (2016)

### Results on practice of EBF among participants

From Table 2, only 109 (27.7%) practiced EBF and 157 (39.9%) still breastfeed baby. In addition, 52 (13.2%) expressed breast milk when away from baby. In conclusion more than half of the participants claimed that their index child has being having episodes of childhood common ailment 260 (66.2%).

**Table 2:** Mode and period of breastfeeding infants by the lactating mothers

Statement	Yes		No	
	Freq	(%)	Freq	(%)
I gave breast milk only for six months	109	27.7	284	72.3
I still breastfeed baby	157	39.9	236	60e1
I expressed breast milk down for baby when away from baby	52	13.2	341	86.8
Episodes of childhood common ailment	260	66.2	133	33.8

Source: Field data, (2016)

Table 3 also showed that the majority, 155 (39.4%) of the respondents-initiated breast feeding within one hour after birth. Again, 195 (49.6%) of the respondents indicated they gave prelateral feeds to their infants during the first six months, 124 (31.6%) indicated not applicable and 74 (18.8%) indicated they give mashed family foods to their infants during the first six months.

**Table 3:** Time of Initiation of breastfeeding and food supplement by the lactating mothers

Response	How soon after delivery did you start breast feeding the baby?	
	Frequency (393)	Percent (%)
Initiation of BF within one hour after birth	155	39.4
Initiation of BF after first one hour after birth	238	60.6
Total	393	100.0
Response	Types of food/drink giving to infants during first six months	
Prelacteal feeds	195	49.6
Mashed family foods	74	18.8
Not applicable	124	31.6
Total	393	100.0
Response	Reasons for giving foods and drinks	
Hot weather makes babies cry; hence they need water	143	36.4
Breast milk production problem	37	9.4
Not able to combine work with breastfeeding	176	44.8
Not applicable	37	9.4
Total	393	100.0
Response	At what age did you start giving other food	
Six months and above	17	4.3
Five months	31	7.8
Four months	33	8.4
Three months	36	9.2
Two months	117	29.8
One month and less	159	40.5
Total	393	100.0

Source: Field data, (2016)

The participants gave a lot of reasons for introducing early feeds to their baby as summaries in the table. Regarding the age at which mothers started giving food to their babies, it was realized that majority had introduced feeds to their baby as early as first (1) and second (2) months respectively. At five months, 31 (7.8%) started taking feeds and at six month, 17 (4.3%) babies also started eating.

### Results on the knowledge of EBF among participants

The result on knowledge as seen in Table 4, majority 277 (70.5%) of the participants had heard about EBF, surprisingly most people choose the electronic media 209 (53.2%) as compared to health professionals 135 (34.4%) as their major source of information. Concerning initiation, the majority concorded that EBP should be started immediately after birth 290 (73.8%). It was also noted that majority believed that EBF should be practice for less than 5 months for a child. The table further summarised the rest of the discriptive findings on the knowledge of the participants on EBF.

**Table 4:** Knowledge of participants about exclusive breasting practice

Variables		Frequency (393)	Percentages (100%)
Have you Heard of EBF	NO	116	29.5
	YES	277	70.5
Majour Source of Knowledge	Health professionals	135	34.4
	Electronic media	209	53.2
	Others	49	12.5
Knowledge on Time to initiate Breastfeeding	Anytime the mother is ready	103	26.2
	Immediately after birth	290	73.8
Duration of EBF	6 months–24 months	49	12.5
	≤5 months	344	87.5
Does EBF improve a child’s immunity?	No	63	16.0
	Yes	330	84.0
Does EBF allows Child spacing?	No	349	88.8
	Yes	44	11.2
Does EBF reduce risk of breast and varian cancer	No	358	91.1
	Yes	35	8.9
Knowledge on how to express breastmilk down for baby	No	350	89.1
	Yes	43	10.9

Source: Field data, (2016)

### Results on the factors associated with EBF

Tables 5 and 6 shows the summary of cross tabulation between EBF, demographic characteristics and some knowledge Characteristic respectively.

**Table 5:** Cross tabulation of EBF and some Sociodemographic predictors of exclusive breastfeeding

Variables		Exclusive breast feeding		Total	Pearson Chi-square value	*P*-values
		No	Yes			
Education of mother	JHS and lower	151	53	204	3.708	.157
	SSS	65	35	100		
	Tertiary	68	21	89		
Total		284	109	393		
Employment Status	Unemployed	96	41	137	.504	.478
	Worker	188	68	256		
Total		284	109	393		
Sex of child	Female	173	54	227	4.177	.041
	Males	111	55	166		
Total		284	109	393		
Age of mother	25 years and below	180	59	239	2.828	.093
	25 years and more	104	50	154		
Total		284	109	393		
Income-Status	Ghc1000 and less (low income)	99	36	135	.554	.758
	Ghc1000–2000 (middle income)	148	61	209		
	Ghc2000 and more (high Income)	37	12	49		
Total		284	109	393		

Source: Field data, (2016)

**Table 6:** Cross tabulation EBF and some knowledge questions

Variables		Exclusive breast feeding		Total	Pearson Chi-square value	*P*-values
		No	Yes			
Does EBF Improves Immunity?	No	40	23	63	2.881	.090
	Yes	244	86	330		
Total		284	109	393		
Does EBF allow child spacing?	No	264	85	349	17.769	<.001
	Yes	20	24	44		
Total		284	109	393		
Does EBF reduce the risk of breast cancer?	No	264	94	358	4.384	.036
	Yes	20	15	35		
Total		284	109	393		

Source: Field data, (2016)

### Discussions of research findings

This research study surveyed a total of 393 mothers from Tamale metropolis, of whom 27.7% reported having exclusively breastfed their infant for the first 6 months of life. The socio-demographic of the participants showed that they all had some level of education. Secondly, most of the lactating women were workers and were middle-income earner who earned between 1000 and 2000 per month. A great majority were matured, they were above 25 years. Literature has shown that there is substantial evidence that breastfeeding women encounter demographic challenges that may affect the initiation and duration of BF. Women who graduated from high school were 70% more likely to breastfeed than those who did not; women who attended college were four times more likely to breastfeed than women who graduated from high school [13]. In a national study in rural Ghana, education, occupation, economic factors and marital status were the factors affecting EB [15]. In contrast, to this finding, an inverse relationship was observed in developing countries. This was possibly due to a perception that ‘breastfeeding was old fashioned’ or an indication of ‘lesser social status’. There is an association between higher education and socioeconomic status, this increases the mother’s ability to purchase infant formula. In addition to educational attainment, maternal age has been identified as among the factors most strongly influencing the initiation, duration and level of infant feeding [16].

It was observed in this study that mothers who were practising EBF were 27.7% which was far below the WHO recommendation of 90% [1]. Also, in a recent study at Tuna, a rural co-district capital of the Sawla-Tuna-Kabla district in the Northern Region of Ghana, a slightly lower rate of 42.0% EBF was realized [17]. These demonstrate a wide gap between the desired and the actual practice of EBF in the study area. The low practices of EBF in this area could be due to low knowledge of EBF, since the study revealed that most mothers did not get information regarding EBF from their health providers. Hence, majority of the mothers were not having knowledge on the need to initiate breastfeeding within early. Majority of the lactating mother had some form of academic education, but this did not translate to their practice of EBF. As seen in the demographic characteristics. The low EBF could be due to lack of time to breastfeed the babies, or the social acceptance and recognition’s given to breast baby feeds or the inadequate knowledge on EBF. Furthermore, the study was carried out on mothers at the antenatal clinics of the hospitals in the Tamale metropolis, a regional capital, and one would have expected them to acquire enough knowledge about EBF and practice EBF, but that was not the cause among the people of Tamale metropolis. In addition, it was also realized that inefficient baby-friendly initiative program might have also been practised at the hospital centers where the study was conducted. Mothers who delivered in baby friendly hospital units are more likely to be knowledgeable about EBF than mothers who did not attend these facilities [17]. A similar observation was also noted in Kware town of Sokoto State of Nigeria where only 31% of the mothers had adequate knowledge of exclusive breastfeeding [18]. Another related study in a similar socio-cultural background also revealed that only 31% of the mothers had adequate knowledge regarding EBF [19]. The knowledge about exclusive breast feeding (EBF) and the practice may depend upon the hospital facilities and their policies in caring for pregnant and lactating mothers after delivery. If there has been a policy to educate pregnant mothers on EBF before and after delivery at various hospitals it would have reflected in their care of the newborns. However, high EBF was noted in some other part of Nigeria where the practice of EBF rate was 75.6% among nursing mothers [20]. It was asserted that the high prevalence of EBF in those studies was because the mothers were mostly workers and took their children with them when they were working, and this makes easier the fostering of EBF practice among mothers. This assertion was also noted by Gladzah, with non-working mothers who were constantly available to breast feed their babies [21]. The Tamale metropolis is in the temperate zone, and because of the sunny and draughty nature of the weather, this might have compiled the mothers to state complementing breastmilk with other liquid diets for the child for six months of age.

Pearson Chi square test of the sociodemographic characteristics associated with EBF showed a significant association between EBF and the sex of the child, X^2^ = 4.177, *P* = .041. Whiles, EBF and the Knowledge on child spacing was X^2^ = 17.769, *P* < .001 and EBF and knowledge on Breast cancer reduction was X^2^ = 4.384, *P* = .036.

This means that mothers who had adequate knowledge about EBF did practice exclusive breastfeeding. This current finding confirms the observation by Mercer on the maternal role attainment theory [22, 23], that stated that in the first stages of “commitment, attachment, and preparation” during pregnancy, the mother acquires the knowledge of breastfeeding and makes a psychological adjustment and prepares for the expectations of her new role. Thus, from the time of birth of infants when motherhood is implicit and understood in the contexts of her social structure, mother’s knowledge of EBF influences the practice [21]. This clearly shows that mothers in this study had not fully achieved this phase of knowledge involved in maternal role identity and EBF decisions making. Similar to this finding [18], opined that knowledge on EBF significantly influenced exclusive breastfeeding among working mothers. In fact, the understanding of the importance of breastfeeding helped mothers adhere to EBF practice.

### Limitations

This study was mainly a cross-sectional study design. Also, the study participants may present recall bias since infants up to 2 years were included. And lastly, from literature reviewed, perception of mothers was one of the factors that affected EBF, but this was not measured in the current study.

## Conclusions

Though majority of the participants had some level of education in the Tamale Metropolis, they had inadequate knowledge about EBF and the rate of EBF was awfully low. Therefore, there was poor knowledge and practice with regards to EBF for the first six months among postpartum mothers in the Tamale Metropolis. These underscore the need to give individual counselling sections to the women at every available opportunity, telling them the importance of EBF to their babies and the women themselves to improve their knowledge about breastfeeding and ultimately their breastfeeding practices.

## Abbreviations

CWC: Child welfare clinic
EBF: Exclusive breastfeeding
TCH: Tamale Central Hospital
TTH: Tamale Teaching Hospital
TWH: Tamale West Hospital
